# The prognostic value of the suPARnostic^® ^ELISA in HIV-1 infected individuals is not affected by uPAR promoter polymorphisms

**DOI:** 10.1186/1471-2334-7-134

**Published:** 2007-11-16

**Authors:** Uffe V Schneider, Rikke L Nielsen, Court Pedersen, Jesper Eugen-Olsen

**Affiliations:** 1 Clinical Research Centre, Copenhagen University Hospital, Hvidovre, Denmark; 2 Department of Infectious Diseases, Odense University Hospital, Odense, Denmark

## Abstract

**Background:**

High blood levels of soluble urokinase Plasminogen Activator Receptor (suPAR) are associated with poor outcomes in human immunodeficiency-1 (HIV-1) infected individuals. Research on the clinical value of suPAR in HIV-1 infection led to the development of the suPARnostic^® ^assay for commercial use in 2006. The aim of this study was to: 1) Evaluate the prognostic value of the new suPARnostic^® ^assay and 2) Determine whether polymorphisms in the active promoter of uPAR influences survival and/or suPAR values in HIV-1 patients who are antiretroviral therapy (ART) naive.

**Methods:**

DNA samples were collected retrospectively from 145 Danes infected with HIV-1 with known seroconversion times. In addition, plasma was collected retrospectively from 81 of these participants for use in the suPAR analysis. Survival was analysed using Kaplan Meier analysis.

**Results:**

Survival was strongly correlated to suPAR levels (p < 0.001). Levels at or above 6 ng/ml were associated with death in 13 of 27 patients within a two-years period; whereas only one of 54 patients with suPAR levels below 6 ng/ml died during this period. We identified two common uPAR promoter polymorphisms: a G to A transition at -118 and an A to G transition at -465 comparative to the transcription start site. These promoter transitions influenced neither suPAR levels nor patient survival.

**Conclusion:**

Plasma suPAR levels, as measured by the suPARnostic^® ^assay, were strongly predictive of survival in ART-naïve HIV-1 infected patients. Furthermore, plasma suPAR levels were not influenced by uPAR promoter polymorphisms.

## Background

Patients with HIV infection receive regular clinical follow-up to assess the need for more invasive treatment regimens such as initiation of ART. Currently, this assessment relies on the evaluation of HIV RNA viral load and CD4 cell count; the former of which requires expensive, high technology laboratory facilities. Thus, there is a need for robust, reliable prognostic markers for HIV infection that can also be used in resource-limited settings. Inflammation increases HIV replication and CD4 T cell depletion [1] and markers of inflammation are potential candidates as prognostic markers for HIV. However, the plasma level of these markers should be independent of genetic polymorphisms in order to be reliable biomarkers.

An elevated level of suPAR is predictive of negative clinical outcome in a number of different diseases such as HIV-1[2], tuberculosis [3], pneumococcal bacteraemia [4], rheumatoid arthritis [5], multiple sclerosis [6] and certain forms of cancer [7,8]. suPAR is an independent marker of survival in HIV-1 infection with a prognostic strength similar to that of CD4+ T-cell count and HIV-1 viral load [2,9].

uPAR is a glycosylphosphatidylinositol (GPI)-linked 3-domain receptor expressed on monocytes, macrophages, activated T-lymphocytes, natural killer cells and neutrophils [10]. uPAR interacts with several molecules including urokinase Plasminogen Activator (uPA), vitronectin and intracellular signalling molecules such as integrins (exp. CD11b/CD18) and a G-coupled receptor [11]. uPAR is involved in several immune functions including cell adhesion and migration (for a review, see [12]). In vitro studies have shown a direct involvement of uPAR/uPA in the late stages of the HIV-1 lifecycle [13,14].

**Figure 1:**
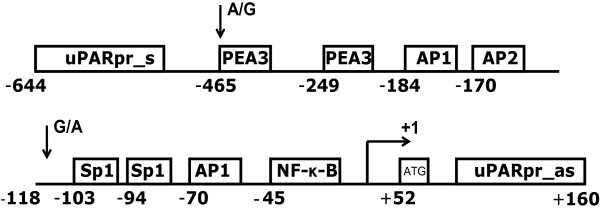
The uPAR promoter from -644 bp to +160 relative to the transcription start site with known regulatory sites and the two identified transitions.

The gene for uPAR maps to chromosome 19q13.2 and consists of 7 exons and 6 introns [15,16]. In vitro studies have located the transcription start site 52 bp upstream to the translation start site (ATG). The promoter activity is primarily restricted to a fragment located -401 bp to +46 bp, with the basal promoter activity restricted to a fragment located -141 bp to +47 bp relative to the transcription start site [17,18]. The promoter contains a number of regulatory sites including AP1, AP2, SP1, NFkB and PEA3 sites (see figure 1) [17-22].

The present study investigates whether plasma suPAR levels measured by the suPARnostic^® ^assay are prognostic for HIV-1 disease progression and whether suPAR levels and survival are influenced by common genetic polymorphisms in the uPAR promoter region.

## Methods

### Patients and sample preparation

The seroconverter cohort consist of 145 HIV-1 infected Caucasians with documented time for seroconversion. DNA was isolated from these individuals using QIAamp Blood Mini Kit according to the manufacturer's protocol (QIAGEN, Valencia, Ca, USA) and stored at -20°C. The cohort comprised 122 males with a mean seroconvertion age of 32.0 years and 23 females who seroconverted at a mean age of 40.2 years. Seroconversion was defined as a seronegative and a seropositive HIV antibody test within a 12 months period. The time of seroconvertion is calculated as the midpoint in time between the last seronegative sample and the first seropositive sample. Patients were followed prospectively every 3–6 months. In this cross-sectional study, one plasma sample from each of the 81 patients taken before the introduction of ART on May 15^th^, 1996 was measured for suPAR (65 males, mean age 38.9 years and 16 females, mean age 44.4 years). Of these 81 patients, 61 patients had a CD4 count included in their medical records within three months of the blood sample date.

### AccuPrime PCR

The AccuPrime PCR (Invitrogen, Carlsbad, Ca, USA) was performed according to the manufactures instructions using 3 μL DNA template and a primermix of 10 μM sense primer (uPARpr_s) 5'-ACTACGCCCGGCTAATTTTT-3' and antisense (uPARpr_as) 5'-CCCCCTCAATTAGACCCTGT-3' (Taq-Copenhagen, Copenhagen, Denmark). PCR-program 94°C for 2 minutes; 30*(94°C for 25 seconds; 55°C for 25 seconds and 68°C for 60 seconds). All PCR-products were run on a 2% volume agarose gel (SeaKem GTG agarose, BMA, Rockland, ME, USA) stained by ethidium bromide with a 100 bp ladder (New England Biolabs, Beverly, MA, USA).

### Sequencing

DNA from 52 individuals was used for sequencing. Prior to sequencing the PCR product was purified using a PCR clean-up-system (Promega, Madison, WI, USA) according to the manufacturers protocol. Sequencing was carried out using 0,5 μL Terminator Ready Reaction Mix (BigDye Terminator v3.1 Cycle sequencing Kit, Applied Biosystems, Foster City, Ca, USA), 3 μL PCR-product, 3pmol Primer (either uPARpr_s or uPARpr_as), 4 μL 5× Sequencing buffer and sterile water to 20 μL. PCR-program: 96°C for 60 seconds; 25*(96°C for 10 seconds, 50°C for 5 seconds, 60°C for 4 minutes) followed by a sodium precipitation and sequencing on an ABI PRISM 3100 Genetic Analyzer (Applied Biosystems).

### Restriction fragment length polymorphisms analysis (RFLP)

A hot start PCR was performed according to manufactures instructions (Qiagen), using 10 μL 10*PCR-buffer (including 15 mM MgCl_2_), 200 μM of each dNTP, 0,2 μM of each primer (uPARpr_s and uPARpr_as), 2.5 units HotStarTaq and 2 μl template DNA per reaction under a PCR-program of 95°C for 15 minutes; 35*(95°C for 30 seconds; 60°C for 30 seconds; 72°C for 1 minute); 72°C for 8 minutes.

Eci-1 restriction analysis was carried out using 1,5 units Eci-1 to 10 μL PCR-product with 1*NEBuffer2 and 100 μg/ml Bovine Serum Albumin (BSA) in a total of 15 μL pr. reaction and a control without Eci-1. MspA1 l restriction analysis was done using 2 units MspA1 l to 10 μL PCR-product with 1*NEBuffer4 and 100 μg/ml BSA in a total of 15 μL per reaction and a control without MspA1 l (New England Biolabs). Following incubation at 37°C for 2 hours, the RFLP product was analysed on 2% agarosegel (SeaKem GTG agarose, BMA) using ethidium bromide staining and a 100 bp ladder (New England Biolabs).

### SuPAR measurements by Enzyme linked immunosorbent assay (ELISA)

SuPAR was measured using the suPARnostic^® ^assay (ViroGates, Lyngby, Denmark) according to the manufactures instructions. Samples were labelled with a number and the technician was blinded to the identity of the patient sample. Briefly, a mastermix of 25 ul plasma and 225 ul assay buffer (containing HRP-labelled secondary antibody) was prepared and 100 ul was added in duplicates onto an ELISA plate (pre-coated from manufactures with catching antibody). After one hour of incubation, the plate was washed, substrate added for 20 minutes and reaction was stopped with 50 ul 0.5 M H_2_SO_4_. Plates were measured at 450 nM with reference 630 nM. One kit measured 39 samples in duplicates in less than two hours. The measured sample intra-assay variation was 3.0 % and inter-assay variation was 10.0 %. This is similar to the inter- and intra-assay variations described in the manufactures kit protocol (1.3 – 4.7% and 1.7 – 5.1%, respectively). The kit standard curve is validated to measure suPAR levels between 0.6 to 22.0 ng/ml with a detection limit (LLOD) of 0.1 ng/ml.

### Statistics

Comparisons were done using the nonparametric Mann-Whitney U test and Pearson Rank test. Heredity of Mentel was tested by the Hardy-Weinberg Equilibrium, and survival of the seroconverters was analyzed using Kaplain-Meier. For the survival analysis, patient follow-up was censored on May 15^th^, 1996 when HAART was introduced to the cohort. The level of significance was set to 0.05. Data were analyzed using the SPSS software, version 13.0.

## Results

### Plasma suPAR measurements and HIV-1 disease progression

SuPAR levels (N = 81) measured by suPARnostic^® ^ranged between 1.22 and 12.16 ng/ml. SuPAR correlated to age, and death during follow-up (table 1).

**Table 1:** Allocation of patients according to suPAR below or above median

	Low suPAR < 4.67 ng/mL (N = 40)	High suPAR ≥ 4.67 ng/mL (N = 41)	Low vs high P (Mann-Whitney U test)
suPAR (ng/ml)*	3,23 (1.22–4.65)	6.96 (4.67–12.16)	
Gender (M/F)	34/6	31/10	NS
			
Eci-1:			
G/G	24	23	NS
G/A	4	5	
A/A	0	0	
			
MspA1 l			
A/A	21	16	NS
A/G	6	10	
G/G	1	2	
			
Age at suPAR meas. (years)*	28.5 (18.8–42.9)	34.5 (19.9–78.2)	< 0.001
Time from seroconversion to suPAR meas. (years)*	0.42 (0.0–4.55)	0.47 (0.0–5.72)	NS
Death within 2 years (Y/N)	0/40	13/28	< 0.001
Median (range) follow-up (years) from sample to 15^th ^May 1996*	5.94 (0.21 – 13.63)	5.38 (0.35 – 10.68)	NS
Death before 15^th ^May 1996 (Y/N)	17/23	25/16	NS

*Median (range). N = number, M/F = Male/Female, NS = non-significant, Y/N = Yes/No

**Figure 2:**
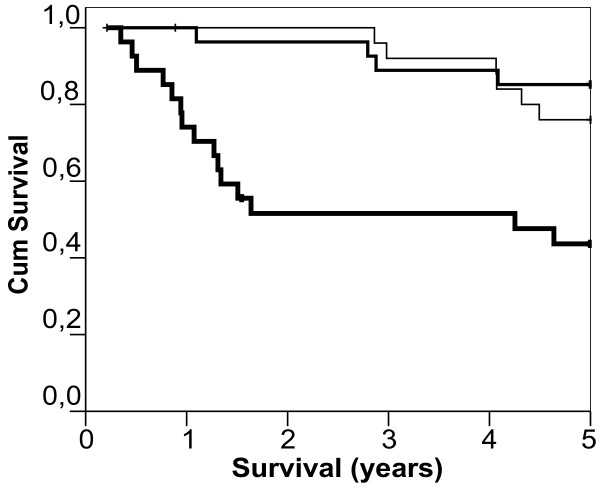
Kaplan Meyer plot of 81 patients divided into tertiles based on suPAR levels. Patients with low suPAR (N = 27, thin line), median suPAR (N = 27, median thick line) and high suPAR (N = 27, thick line).

**Figure 3:**
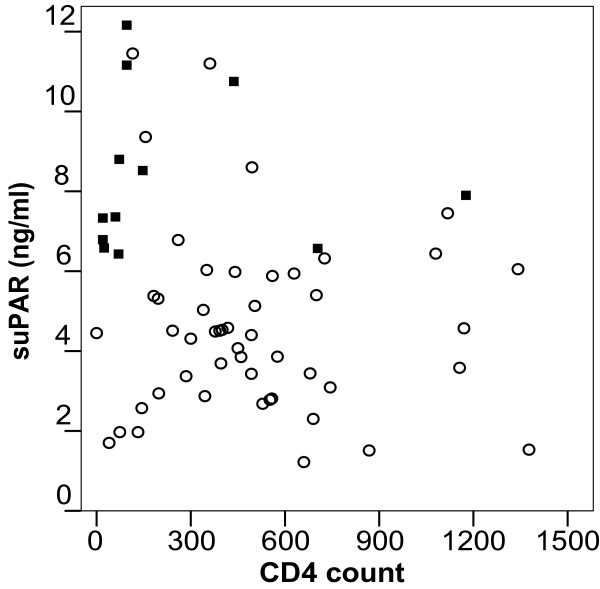
Scatterplot of CD4 T cell counts and suPAR. No significant correlation was observed (Pearson r = -0.22, p = 0.09). Patients that died (N = 13) within 2 years of blood sampling are shown as black squares.

For the Kaplan Meier analysis, patients were divided into three equally sized groups of 27 patients based on their respective suPAR levels. As shown in figure 2, patients with low suPAR (suPAR range 1.22–3.85 ng/ml) and those with median suPAR levels (suPAR range 3.86–5.88) progressed significantly slower to death compared with patients with high suPAR levels (suPAR range 5.94–12.16)(p < 0.001). Scatter plot of CD4 count against suPAR level shows no significant correlation (Pearson r = -0.22, p = 0.09) (figure 3).

### uPAR promoter restriction fragment length polymorphism assay

**Figure 4:**
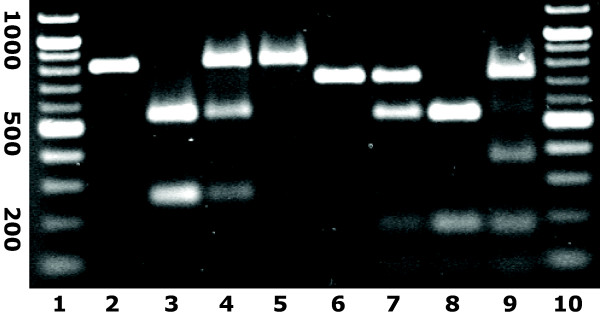
Restriction pattern by Eci-1 and MspA1 l fragmentation of the uPAR promoter sequence. Lane 1 and 10, 100 bp-marker; lane 2 non digested PCR-product, lane 3–5 Eci-1 fragmentation of the -118 G to A transition (G/G; G/A and A/A), lane 6–8 MspA1 l fragmentation of the -465 A to G transition (A/A; A/G and G/G); lane 9 Eci-1 and MspA1 l fragmentation of a double heterozygote individual.

The uPAR promoter was sequenced in 52 individuals covering the active promoter from -643 bp upstream to +159 bp downstream of the transcription start site. Two mutations were detected: a G to A transition at -118 and an A to G transition at -465 upstream of the transcription start site. Of the 52 individuals, 43 (≈ 83%)were found to be -118 G/G, 8 (≈ 15%) to be G/A and 1 (≈ 2%) to be A/A. For the -465 transition, 31 (≈ 60%) were A/A, 20 (≈ 38%) were A/G and 1 (≈ 2%) was G/G. It was possible to develop a RFLP assay based on the sequencing results. By Eci-1 endonuclease fragmentation the -118 mutant A can be separated from the wildtype G by cutting of the A allele between base pair -106 and -107. The wildtype A and the mutant G at -465 can be differentiated by MspA1 l, due to a restriction site between base pair -466 and -467 in the mutant allele (figure 4). The results obtained by RFLP were in concordance with the sequencing results.

### uPAR promoter transitions and HIV-1 disease progression

To investigate whether the uPAR promoter transitions influence HIV disease progression, the entire seroconverter cohort (N = 145) was analyzed for the promoter transitions using the RFLP assay. In this cohort, 130 (≈ 90%) were -118 G/G carriers and 15 (≈ 10%) patients were G/A heterozygotes while no homozygous A/A carriers were found. With regard to the -465A/G transition, 84 (≈ 58%) were A/A, 55 (≈ 38%) were A/G heterozygotes and 6 (≈ 4%) were G/G homozygotes. The mutations were in Hardy-Weinberg Equilibrium. No individuals were homozygotes for one of the transitions and heterozygote for the other transition, whereas a total of six individuals were heterozygotes for both transitions. By RFLP with MspA1 l and Eci-1 on PCR-products from these six individuals, fragments matching a restriction pattern of 709, 348, 184/177 and 85 bp were identified. This is consistent with one of the alleles not to be restricted by Eci-1, while the other allele additionally is restricted by MspA1 l, indicating that the two polymorphism are not travelling on the same allele and thereby are in genetic disequilibrium (see figure 4).

**Figure 5:**
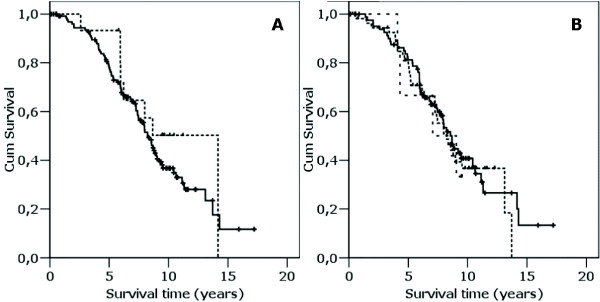
**a-b**. Kaplan-Meier analysis of promoter transitions. a) for the -118 G to A transition (— G/G; ---- G/A), b) for the -465 A to G transition (— A/A; ------ A/G; - - - G/G). + indicate censored.

Mann Whitney analysis on difference in suPAR levels between carriers of the different genotypes showed no significant difference in suPAR plasma levels (p > 0.5). Similar, the distribution of individuals with different genotypes according to median suPAR was not significant (table 1). Finally, using Kaplan Meier analysis, no correlation between survival and uPAR promoter transitions was found (figure 5a and 5b).

## Discussion

The aim of the current study was to evaluate the prognostic value of the newly available suPARnostic^® ^assay in a cohort of HIV infected patients who were ART naïve. Furthermore, this study was designed to assess whether suPAR values, as measured by the new assay, are influenced by polymorphisms in the active region of the uPAR promoter.

We hypothesize that elevated plasma suPAR levels indicate a heightened inflammatory state. SuPAR correlates to C-reactive protein [5], which also predicts survival rates in HIV-1 infected individuals [23]. Inflammation and immune activation are believed to be drivers of HIV replication and CD4 T-cell depletion [24,25].

In agreement with the inflammation hypothesis, previous studies have shown a negative correlation between suPAR levels and CD4 T-cell counts and a positive correlation between suPAR and HIV viral load [2,9] Likewise, we observed a negative correlation between suPAR levels and CD4 count in the present study, although it did not reach significance, which is likely due to the small number of patients included in the present study. In agreement with previous studies on suPAR and HIV-1, it appears that both suPAR levels and CD4 T-cell counts are independent predictors of mortality in HIV infection.

To further explore the combined prognostic value of suPAR and CD4, we plotted patients who died within a two-year follow-up period in a CD4/suPAR graph. Patients with a low CD4 count (defined as below 200) and high suPAR levels (defined as above 6 ng/ml) had high two-year mortality while those with low CD4 and low suPAR levels were still alive after two years. Thus, suPAR and CD4 both added clinical value on the risk of mortality in this cohort of non-ART-treated HIV-1 patients.

A cut-off limit for suPAR has yet to be defined. In this study, only 1 of 54 patients with plasma suPAR levels below 6 ng/ml died within two years following blood sampling, while 13 of 27 patients with suPAR at or above 6 ng/ml died in the same period (figure 3). We focused the analysis of suPARs prognostic value to a two-year period of the following two reasons: 1) Disease progression biomarkers, including CD4 and viral load are measured at regular intervals (3 – 6 months) to monitor the state of the patient. Similarly, other biomarkers, such as suPAR, should provide useful and immediate prognostic clinical information 2) Due to the small number of patients included in the present study, the numbers were too small for meaningful statistical analysis following this period. Hence, larger studies are needed to confirm whether 6 ng/ml is a relevant cut-of suPAR and longitudinal studies should address the dynamics of suPAR in relation to HIV disease progression.

Two common mutations were defined in this study: a G to A transition at -118 and an A to G transition at -465 upstream of the transcription start site. Neither of these mutations had any influence on survival nor suPAR plasma levels. Previous studies have shown that in vitro regulation of the PEA3/etc site at -247 to -241 could influence the promoter activity [18,22], however these studies did not analyze plasma suPAR levels.

This is the first paper that applies the suPARnostic^® ^assay to non-ART HIV disease progression. A resent South African study employed the suPARnostic^® ^assay to individuals initiating ART [26]. In 293 HIV infected initiating ART with a median CD4 count of 47 cells per ul and followed for 4 months, pre-treatment CD4 and suPAR levels were predictors of mortality (39 died) during the 4 month treatment period. In multivariate analysis, only suPAR levels remained significantly associated with survival (RH per log suPAR increase 10.0, 95%CI 2.8 – 35.7) [26].

## Conclusion

This study confirms that the suPAR level measured using the suPARnostic^® ^assay is an independent prognostic marker of mortality in HIV-1 patients who are not on ART treatment. Moreover, these results suggest that patients with suPAR levels at or above 6 ng/ml should be considered for initiation of ART, in particular in cases with CD4 between 350 and 200 cells per ul. Finally, this study found that the value of suPAR levels as a biomarker for survival in HIV patients is not affected by uPAR promoter polymorphisms.

## Competing interests

JEO is founder of, and shareholder in, ViroGates. ViroGates IP includes patents on using suPAR for diagnostic and prognostic purposes.

## Authors' contributions

UVS, RLN and JEO designed the study and analysis. UVS and RLN did the laboratory work. CP established the HIV-1 cohort. UVS and JEO wrote the first draft. All authors read and approved the final manuscript

## Pre-publication history

The pre-publication history for this paper can be accessed here:


